# Different *Cis*-Regulatory Elements Control the Tissue-Specific Contribution of Plastid ω-3 Desaturases to Wounding and Hormone Responses

**DOI:** 10.3389/fpls.2021.727292

**Published:** 2021-10-27

**Authors:** María A. Luján, Ángel Soria-García, Ana Claver, Patricia Lorente, María C. Rubio, Rafael Picorel, Miguel Alfonso

**Affiliations:** Estación Experimental de Aula Dei (EEAD-CSIC), Avenida Montañana, Spain

**Keywords:** *Arabidopsis thaliana*, desaturase, trienoic fatty acid, hormone, defense

## Abstract

Trienoic fatty acids are essential constituents of biomembranes and precursors of jasmonates involved in plant defense responses. Two ω-3 desaturases, *At*FAD7 and *At*FAD8, synthetize trienoic fatty acids in the plastid. Promoter:GUS and mutagenesis analysis was used to identify *cis*-elements controlling *AtFAD7* and *AtFAD8* basal expression and their response to hormones or wounding. *AtFAD7* promoter GUS activity was much higher than that of *AtFAD8* in leaves, with specific *AtFAD7* expression in the flower stamen and pistil and root meristem and vasculature. This specific tissue and organ expression of *AtFAD7* was controlled by different *cis*-elements. Thus, promoter deletion and mutagenesis analysis indicated that WRKY proteins might be essential for basal expression of *AtFAD7* in leaves. Two MYB target sequences present in the *AtFAD7* promoter might be responsible for its expression in the flower stamen and stigma of the pistil and in the root meristem, and for the *AtFAD7* wound-specific response. Two MYB target sequences detected in the distal region of the *AtFAD8* gene promoter seemed to negatively control *AtFAD8* expression, particularly in true leaves and flowers, suggesting that MYB transcription factors act as repressors of *AtFAD8* gene basal expression, modulating the different relative abundance of both plastid ω-3 desaturases at the transcriptional level. Our data showed that the two ABA repression sequences detected in the *AtFAD7* promoter were functional, suggesting an ABA-dependent mechanism involved in the different regulation of both ω-3 plastid desaturases. These results reveal the implication of different signaling pathways for the concerted regulation of trienoic fatty acid content in Arabidopsis.

## Introduction

Glycerolipids and fatty acids are building blocks of cell membrane-forming bilayers, providing the barrier for cell compartmentalization and the matrix in which many membrane proteins, like photosynthetic complexes in the chloroplast or the respiratory chain in the mitochondria, perform their function. The glycerolipid content and the degree of unsaturation of their fatty acids, determine the physical-chemical properties of bio-membranes, conferring the optimal fluidity that allows the motion of proteins and molecules within the membrane (Chapman, 1975). These dynamic properties influence the function of many proteins, but also determine some acclimation responses to changes in environmental conditions. Thus, changes in trienoic fatty acid (TA) content are associated with temperature acclimation responses (reviewed in Iba, 2002; Guschina and Harwood, 2006). In addition, plastid TAs are precursors for the biosynthesis of jasmonates (JAs) (Vick and Zimmerman, 1984) which are key regulators of defense responses against pathogen or herbivore attack (McConn et al., 1997; Devoto and Turner, 2003; Farmer et al., 2003; Lorenzo and Solano, 2005). Besides this role, JAs are also involved in plant developmental processes such as root growth and pollen maturation (Feys et al., 1994; McConn and Browse, 1996).

In plants, TAs are synthesized from dienoic fatty acids (DAs) by the activity of ω-3 fatty acid desaturases, which are integral membrane enzymes located in two different cell compartments; FAD3 is localized in the endoplasmic reticulum (ER) (Dyer and Mullen, 2001) while FAD7 and FAD8 are plastid specific (Browse et al., 1986; Andreu et al., 2007; Román et al., 2015). In Arabidopsis, single nuclear genes encode for each of these enzymes (Yadav et al., 1993; Gibson et al., 1994). In other plant species, like soybean, several isoforms of *Gm*FAD3, *Gm*FAD7 and *Gm*FAD8 ω-3 desaturases have been detected (Bilyeu et al., 2003; Andreu et al., 2010; Román et al., 2012). In Arabidopsis, *At*FAD7 and *At*FAD8 desaturases share a high percentage of identity (>85%), (Gibson et al., 1994; Román et al., 2015). Despite their similar enzymatic activity and subcellular localization, both ω-3 desaturases do not act in a similar manner. Functional analysis in Arabidopsis indicated that both proteins differed in their acyl-group chain length and lipid head group specificities (Román et al., 2015). Recent tissue distribution analysis showed that *At*FAD7 seemed to be the major ω-3 plastid desaturase in leaves (Soria-García et al., 2019). This higher *At*FAD7 abundance was consistent with a higher gene expression (transcript and promoter:GUS activity), suggesting that the modulation of the relative abundance of both plastid ω-3 desaturases was exerted primarily at the transcriptional level (Soria-García et al., 2019).

The non-redundant role of both plastid ω-3 desaturases is not only related to their relative abundance or substrate specificity but also to their different participation in stress responses to developmental or environmental stimuli. Thus, *FAD7* transcripts increased after wounding in Arabidopsis, tobacco or soybean (Nishiuchi et al., 1997; Reymond et al., 2000; Matsuda et al., 2009; Andreu et al., 2010) without changes in *FAD8* gene expression (Andreu et al., 2010). Conversely, cold temperatures increased *FAD8* mRNA specifically with a decrease of *FAD7* mRNA in Arabidopsis or maize (Gibson et al., 1994; Berberich et al., 1998; Román et al., 2015). Although the expression profiles of genes encoding fatty acid desaturases and their modulation by environmental signals have been widely reported in the literature, very few data are available of their promoter structure and the identification of *cis*-regulatory elements and transcription factors (TFs) controlling ω-3 desaturase expression in plants. In fact, very few examples of TFs involved in the control of fatty acid biosynthesis and modification have been reported in the literature. The most characterized one is WRINKLED1 (WRI1), which belongs to the APETALA TF family that controls fatty acid biosynthesis specifically in seeds (Cernac and Benning, 2004; Baud et al., 2007; Kong and Ma, 2018). More recently, certain members of the MYB family, like MYB89 and MYB96 have been involved in the positive or negative regulation of genes involved in fatty acid biosynthesis during seed development (Wang et al., 2014; Li et al., 2017; Lee et al., 2018). Regarding fatty acid desaturases, bZIP67, a leucine zipper protein, was identified to activate ER FAD3 desaturase during seed maturation in Arabidopsis (Mendes et al., 2013). All these cases were related to seed oil metabolism. However, very little information is available of the control of ω-3 desaturase activity in other tissues like leaves, where TAs represent more than 80% of total fatty acids (Román et al., 2015). In the case of *FAD7*, heterologous expression in tobacco of an 825 bp *AtFAD7* promoter:GUS fusion showed that its expression was restricted to tissues containing chloroplasts (Nishiuchi et al., 1999). Interestingly, wound treatments induced promoter-driven GUS activity in other tissues like stems or roots (Nishiuchi et al., 1999). Further analysis identified two regions in the promoter involved in the leaf or root wound-response of *AtFAD7*, suggesting different regulation of the wound response of *AtFAD7* between both tissues (Nishiuchi et al., 1999). In the case of *AtFAD8*, protein stability has been signaled as a mechanism involved in the control of desaturase activity in response to temperature (Matsuda et al., 2005). However, the differences in transcript abundance between both plastidial desaturases (Soria-García et al., 2019) as well as the cold-specific induction of the *AtFAD8* mRNA (Gibson et al., 1994; Berberich et al., 1998; Román et al., 2015) also point to differences in transcription as a key mechanism for the control of the basal activity of both desaturases. Unfortunately, the *cis*-regulatory elements and TFs involved in the basal expression of *AtFAD7* and *AtFAD8* genes and their different tissue-specific expression or response to biotic or abiotic stresses remain unknown.

As a first step to identify *cis*-regulatory elements and TFs involved in the control of *AtFAD7* and *AtFAD8* genes and their non-redundant activity, a functional characterization of the promoters from both genes was carried out by promoter:GUS fusions. Deletion analysis of these promoter sequences, together with site-directed mutagenesis of specific target sequences, allowed us to identify *cis*-regulatory elements involved in the control of the basal expression of both genes in the different plant tissues and organs, and in their specific responses to hormones or wounding. Our results show that different TF families may be involved in the control of the different abundance of both plastidial ω-3 desaturases and their different response to hormone and defense signaling pathways for the control of plastid TA biosynthesis in Arabidopsis.

## Materials and Methods

### Plant Material and Growth Conditions

All plant lines obtained in this work were derived from *Arabidopsis thaliana* Col-0 line. Arabidopsis seeds were sterilized and germinated in MS medium or directly in pots. Seeds were vernalized for 3 days at 4°C in darkness and then moved to a growth chamber. Growth conditions were light intensity of 120-150 μmol.m^–2^. s^–1^, with a 16h/8h light/darkness photoperiod, at 20-22°C and a relative humidity of 45%. Depending on the experiments, roots and rosette leaves of 14-day old plants were gathered for GUS staining experiments or stored at –80°C for further qPCR analysis. For flower analysis, plants were grown in the chamber for 4-5 weeks until complete flowering.

### Experimental Treatments

For wounding treatments, 2-week old plants were wounded by pressing the leaf with a pipette tip. Plants with the wounded leaves were kept in the growth chamber for an additional hour. Wounded and control unwounded leaves were rapidly incubated with the GUS reactive or frozen in liquid nitrogen for further analysis. For ABA treatments, plants were grown on Whatman filter paper in MS plates for 12 days until both cotyledonal and true leaves were fully developed. Then, the filter papers containing the grown plantlets were transferred to MS plates containing 100 μM (±) ABA (Sigma) for 48 h before performing GUS or gene expression analysis. ABA was dissolved in methanol. Methanol was used for mock treatments in the ABA experiments. Expression of *ABI1* (At4g26080) gene was analyzed to monitor the effect of the ABA. For MeJA treatment, 2 week-old plants were sprayed with 100 μM methyl jasmonate (MeJA, SIGMA) for 2 h, before being analyzed for GUS histochemical activity. Analysis of variance (ANOVA) was applied to compare treatments. Statistical analyses were carried out with the program Statgraphics Plus for Windows 2.1, using a level of significance of 0.05.

### Generation of Promoter: GUS Arabidopsis Lines

Generation of transgenic lines expressing the 1,682 and 2,958 bp *AtFAD7* and *AtFAD8* promoter fragments, respectively, fused to GUS was described in Soria-García et al. (2019). The different promoter deletion fragments used in this work were generated by PCR using Phusion High-Fidelity DNA Polymerase (Thermo) and specific primers (Supplementary Table 1). In the case of the 1,682 bp *AtFAD7* promoter sequence, three deletions were obtained. The first one was a 994 bp fragment corresponding to the distal region of the promoter and devoid of all the elements present in the proximal regions of the promoter. The second one corresponded to a 703 bp fragment of the proximal region of the promoter that was devoid of several putative *cis* regulatory elements located in the distal regions. Finally, a shorter 499 bp deletion was obtained from this proximal fragment. In the case of the *AtFAD8* 2,958 bp promoter, a 1,061 distal promoter fragment was generated, devoid of all the elements located in the proximal regions of the promoter. A second fragment of 1,912 bp, that contained the rest of the proximal promoter sequence was obtained. Two further deletions of 643 and 290 bp, respectively were also generated from this proximal fragment, searching for the selective elimination of specific putative *cis* regulatory elements identified in the *in silico* analysis. All PCR amplification products were cloned in a pENTR D-TOPO entry vector. *AtFAD7* and *AtFAD8* putative promoter fragments, now flanked by the appropriate AttL sites, were sub-cloned in a pMDC163 plasmid (Curtis and Grossnicklaus, 2003) through Gateway technology^®^, using LR Clonase II enzyme mix (Invitrogen). All cloning products were sequenced to confirm the absence of PCR errors. Agrobacterium-mediated transformation (GV3101 strain) of Arabidopsis plants was performed by floral dip method (Clough and Bent, 1998). Positive transformants were selected for hygromicine resistance and genotyped by Phire^®^ Plant Direct PCR Kit (Thermo). Homozygote lines T3 were segregated for further analysis. At least 10 independent transgenic lines were obtained for each event and three of them were analyzed.

### Site-Directed Mutagenesis of Specific Target Sites From Both *AtFAD7* and *AtFAD8* Promoters

Site-directed mutagenesis of specific sequences from both *AtFAD7* and *AtFAD8* promoter fragments was performed using the QuickChange XL^®^ site-directed mutagenesis kit from Agilent. Both the 1,682 and 2,958 bp fragments already cloned in the pMDC163 vector were used as template for mutagenesis of the *AtFAD7* and *AtFAD8* promoters, respectively. Primers used for mutagenesis are listed in Supplementary Table 1.

### Histochemical and Fluorometric GUS Assays

GUS staining protocol was adapted from Jefferson et al. (1987) as described in Soria-García et al. (2019). Samples were visualized in a Leica M165 FC stereomicroscope. Results shown are representative of 3-6 individual plants of at least two transformation events. GUS fluorometric protocol was adapted from Vitha et al. (1993) as reported in Soria-García et al. (2019). Fluorescence of aliquots was measured using a Synergy^TM^ HT plate reader (BioTek) at 365 nm excitation wavelength and 455 nm emission wavelength. Results shown are representative of at least three biological samples of at least two transformation events.

### Quantitative PCR Analysis

Total RNA was extracted from 0.5 g of rosette leaves and 0.1 g of roots with Trizol (Life Technologies) according to manufacturer’s instructions. First-strand cDNA was synthesized from 3 μg of DNAase-treated RNA with M-MLV reverse transcriptase (Promega) and oligo dT. Quantitative PCR (qRT-PCR) was performed using a 7,500 Real Time PCR System (Applied Biosystems), SYBR Green Master Mix (Applied Biosystems), and specific primers (Supplementary Table 1). The Ct values were calculated relative to EF1α reference gene (*At5g60390*) using 2^–ΔΔCt^ method (Livak and Schmittgen, 2001). EF1α expression was stable in all the conditions tested. Data were obtained from the analysis of at least three biological samples with three independent technical repeats for each sample.

### *In silico* Analysis of Plastid ω-3 Fatty-Acid Desaturase Promoters

*In silico* analysis of upstream sequences relative to *AtFAD7* (*At3g11170*) and *AtFAD8* (*At5g05580*) genes was made using three different on-line platform tools: Plant *Cis*-Acting Response Elements software (PlantCare) (Lescot et al., 2002); Plant *Cis*-Acting Regulatory DNA Elements (PLACE) (Higo et al., 1999) and MOTIFSAMPLER^1^ (Claeys et al., 2012). Phylogenetic trees were generated with the PHYML software (Guindon and Gascuel, 2003; www.phylogeny.fr) with bootstraps 500.

## Results

### Functional Analysis of the *AtFAD7* Gene Promoter Using Promoter:GUS Fusions

The *AtFAD7* promoter used in this work corresponded to the 1,682 bp upstream sequence of the *AtFAD7* (*At3g11170*) gene, previously characterized by our group (Soria-García et al., 2019). The expression and activity of the *AtFAD7* promoter sequence used in that work correlated well with the expression of the endogenous *AtFAD7* gene in leaves and roots, suggesting that most, if not all, of the elements controlling the transcription of *AtFAD7* were present in the 1,682 bp fragment (Soria-García et al., 2019). In this work, the 1,682 bp *AtFAD7* promoter sequence was studied into detail and the distribution of putative *cis*-elements analyzed by searching PLACE^2^), PLANTCARE^3^ and MOTIFSAMPLER^4^ webtools. This 1,682 bp fragment doubled the 824 bp one used by Nishiuchi et al. (1999) for the heterologous expression of the *AtFAD7* gene in tobacco. This higher length was justified by the presence of several significant putative *cis*-acting elements identified by the bioinformatics analysis. Thus, a putative TATA box was located at -182 with respect to the ATG, near the transcription start point (TSP) located at -155 of the ATG of the *AtFAD7* gene. Two putative MYB target sequences were detected at -1,093/-1,087 (CAACTG) and -927/-922 (TAACGG) with respect to the ATG (Table 1). These MYB target sequences corresponded well with the core-binding sequence 5’-TAACTG-3’ described for *At*MYB2 (Urao et al., 1993). The reverse complement of these two MYB sequences (as present in the negative strand) corresponded well to the MYB consensus sequence 5′-CNGTTA/G-3′ determined by Romero et al. (1998) or that of MYB44 in the WRKY70 promoter (Shim et al., 2013). Three WRKY consensus sequences (Rushton and Somssich, 1998; Rushton et al., 2010), were located at -594/-588, -521/-516 (TTGACT) and -263/-258 (TTGACC), with respect to the ATG (Table 1). These WRKY boxes fully corresponded to the functional sequence that has been experimentally determined to bind WRKY proteins (Ciolkowski et al., 2008). W boxes are usually found in clusters in many stress-inducible promoters (Maleck et al., 2000). This seemed to be also the case in the *AtFAD7* promoter. Two ABA repression sequences, CAACTTG and GAAGTTG (Wang et al., 2011), placed at -278/-267 and -203/-197 with respect to the ATG in the *AtFAD7* promoter sequence were detected (Table 1). These sequences, which are one of the scarce ABA-repression elements identified up to now, are present in most ABA-repressed genes (Wang et al., 2011). Their position, flanking the TATA and the TSP of the *AtFAD7* gene, suggested that they could be acting as negative effectors. Other regulatory elements, as G boxes (Siberil et al., 2001), were also detected in the *AtFAD7* promoter sequence (Table 1).

**TABLE 1:** List and positions in the *Arabidopsis thaliana AtFAD7* and *AtFAD8* promoters of the different putative cis-regulatory elements identified in the *in silico* analysis.

**Promoter**	**Putative cis regulatory element**	**sequence**	**position**
*AtFAD7*	MYB	CAACTG	−1,093/−1,087
(1,682 bp)		TAACGG	−927A922
	WRKY	TTGACT	−594A588
		TTGACT	−521/−516
		TTGACC	−263A258
	CBF/DREB	AGCGAC	−737/−731
	G-box	ACGT	−1,461/−1,457
		ACGT	−1,341/−1,337
		ACGT	−1,060/−1,056
		ACGT	−527A523
	ABA repression	CAACTTG	−278/−271
	Sequence	GAAGTTG	−203/−197
*AtFAD8*	MYB	CAACTG	−2,840/−2,834
(2,958 bp)		CAACGG	−2,708/−2,703
	WRKY	TTGACC	−490/−484
		TTGACT	−428A423
		TTGACT	−309/−303
	CBF/DREB	GCCGAC	−1,300/−1,294
		GCCGAC	−1,297/−1,292
		GCCGAC	−346/−340
		GCCGAC	−128/−122
		GCCGAC	−125/−120
		GCCGAC	−122/−117
	MYC/ICE1	CAAATG	−1,503/−1,498
		CAAATG	−652A647

*All the sequences have been described to be functional in different analysis: MYB, (Romero et al., 1998; Shim et al., 2013); WRKY, (Rushton and Somssich, 1998); ABA repression sequences, (Wang et al., 2011); G-boxes, (Siberil et al., 2001); CBF/DREB, (Thomashow, 1999) and MYC/ICE1, (Yamaguchi-Shinozaki and Shinozaki, 2006).*

**FIGURE 1:**
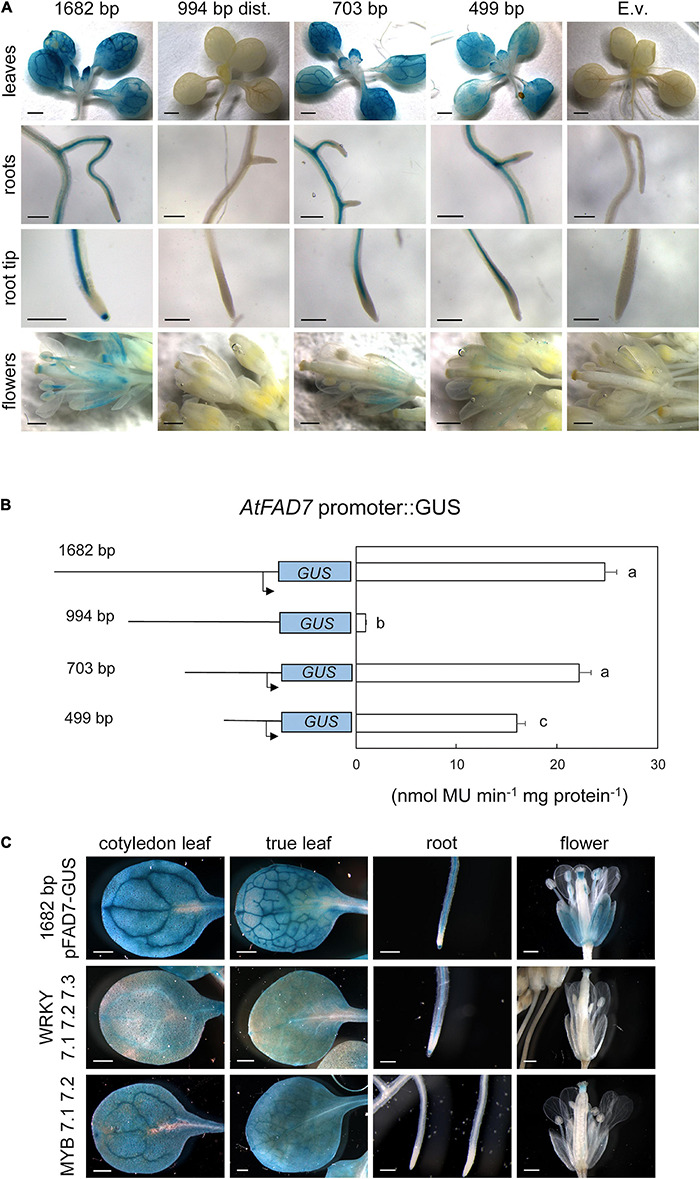
GUS histochemical localization of the *AtFAD7* gene promoter. **(A)** *AtFAD7* promoter:GUS staining in leaves, roots and flowers. Results are shown for the control *AtFAD7* 1,682 bp fragment and the three deletions of 994 bp, 703 bp, and 499 bp, respectively. E.v. shows results obtained with the empty vector. Results were obtained after 1 h of GUS staining. Images are representative of at least three independent transgenic lines. Scale bars represent 1,000 μm in plantlets, 200 μm in roots and 500 μm in flowers. **(B)** GUS activity in the *AtFAD7* gene promoter fragments. Activity was determined in extracts from rosette leaves of 2-week old plants carrying the different *AtFAD7* promoter fragments. Results were obtained after 1 h of GUS staining. A schematic diagram of the different promoter length fragments used for the analysis is also shown. Activity was determined as nmol MU⋅min^–1^ ⋅mg protein^–1^. Data are means ± SE from at least five independent determinations of three independent transgenic plants. Different letters indicate significant differences among treatments (*P* < 0.05) **(C)** *AtFAD7* promoter:GUS staining in leaves, roots and flowers in transgenic lines in which the three WRKY or the two MYB putative target sequences detected in the *AtFAD7* gene 1,682 bp promoter fragment were modified by site-directed mutagenesis. WRKY 7.1 7.2 7.3 and MYB 7.1 7.2 represents the lines carrying the site-directed mutation on the WRKY and MYB target sequences, respectively. Lines containing the 1,682 bp *AtFAD7* fragment were used as a control in all tissues. Results were obtained after 1 h of GUS staining. Images are representative of at least three independent transgenic lines. Scale bars represent 500 μm in leaves and flowers, 200 μm in roots.

To analyze the functionality of the *cis*-acting elements identified by the *in silico* analysis, three deletions of the 1,682 bp *AtFAD7* promoter sequence were obtained by PCR (Supplementary Table 1). Several T3 Arabidopsis transgenic lines carrying the different promoter:GUS fusions were further analyzed for GUS activity. As reported previously (Soria-García et al., 2019), high GUS histochemical staining was detected upon 1 h staining on leaf tissue including mesophyll cells and vasculature in either cotyledonal, or rosette leaves from plants harboring the 1,682 bp *AtFAD7* promoter fragment (Figure 1A). No detectable GUS activity was observed in leaves from control transgenic plants expressing the empty pMDC163 vector (Figure 1A). Plants harboring the deleted promoter fragments were also analyzed. Lines harboring the 994 bp distal fragment showed no GUS staining in leaves when compared to the control 1,682 bp lines (Figure 1A), suggesting that the regulatory elements present in this distal promoter fragment were not capable to drive alone the expression of the *AtFAD7* in leaves. However, although TATA boxes were identified in this portion of the sequence, it cannot be discarded that some essential element for basal transcription could have been eliminated in this fragment. When the 703 bp fragment, that contained the proximal region of the promoter, was assayed, the GUS staining pattern was nearly identical to that of the 1,682 bp control one (Figure 1A), indicating that elements in the *AtFAD7* promoter placed in the 703 bp fragment were sufficient to drive the basal expression of the GUS gene at control values in leaves. A decrease in total GUS staining was observed in rosette leaves when the 499 bp fragment was analyzed (Figure 1A).

GUS activity was determined in rosette leaves using umbelliferol to confirm the GUS histochemical data. The 1,682 bp *AtFAD7* promoter sequence showed GUS activity of 24.7 nmol MU⋅min^–1^⋅mg protein^–1^. These activity values were similar to those reported by Nishiuchi in the expression of their 825 bp fragment in tobacco (Nishiuchi et al., 1999). Again, the 994 bp distal promoter fragment showed very low GUS activity (less than 2 nmol MU⋅min^–1^⋅mg protein^–^1; Figure 1B). The 703 bp promoter fragment showed a GUS activity of 22.23 nmol MU⋅min^–1^⋅mg protein^–1^, a 90% of that obtained with the 1,682 bp *AtFAD7* promoter sequence (Figure 1B). The 499 bp fragment showed a substantial reduction of GUS activity (16.03 nmol MU⋅min^–1^⋅mg protein^–1^) but still retained a 65% of the control one (Figure 1B).

Functional analysis of the *AtFAD7* gene promoter in non-photosynthetic tissues was also performed. Our data showed intense GUS staining of the 1,682 bp promoter fragment in the root vasculature from both primary and secondary roots and particularly in the root meristem (Figure 1A). This result contrasted with that obtained by Nishiuchi et al. (1997) that restricted *FAD7* promoter activity to chloroplast containing tissues in unwounded plants. The 994 bp distal promoter deletion fragment showed no GUS staining (Figure 1A), similar to that obtained with the empty vector (Figure 1A). Transgenic lines harboring the 703 bp and 499 bp proximal fragments showed GUS staining in the root vasculature of the primary root (Figure 1A), similar to that obtained with the 1,682 bp promoter sequence. However, no GUS staining was detected in the root meristem when compared to the 1,682bp *AtFAD7* promoter fragment (Figure 1A). The results suggested that the elements responsible for *AtFAD7* expression in the root vasculature are different from those responsible for its expression in the root meristem, that might be located in the distal part of the promoter, upstream the 703 bp fragment.

In flowers, the 1,682 bp *AtFAD7* promoter fragment produced high GUS staining in the filament of the stamen and in the stigma of the pistil (Figures 1A,C). High GUS staining was also detected in sepals (green tissue) but not in petals (Figures 1A,C), consistent with the GUS histochemical activity detected in leaves. As it happened in leaves and roots, no GUS staining in these organs was detected when the 994 bp distal fragment of the promoter was analyzed (Figure 1A). Interestingly, no GUS staining was detected in the stigma or the filaments of the stamen when the 703 bp fragment was tested, although it maintained some staining in the sepals (Figure 1A). Further deletion of the promoter in the 499 bp fragment drastically decreased GUS staining to almost undetectable levels in all flower organs (Figure 1A). These results suggested that elements that participate in the expression of the *AtFAD7* gene in anthers and the pistil of the ovarium in flowers are located upstream the 703 bp fragment and, accordingly, may be different to those that participate in the control of the expression in leaves and the root vasculature. No GUS staining was detected in seeds from the transgenic lines expressing the 1,682 bp *AtFAD7* (Supplementary Figure 1). Interestingly, GUS histochemical activity was detected in both extremes of the pod specifically in the case of the *AtFAD7* gene promoter (Supplementary Figure 1). This specific GUS activity might be related with that observed in the stigma of the flower pistil in these same lines.

**FIGURE 2:**
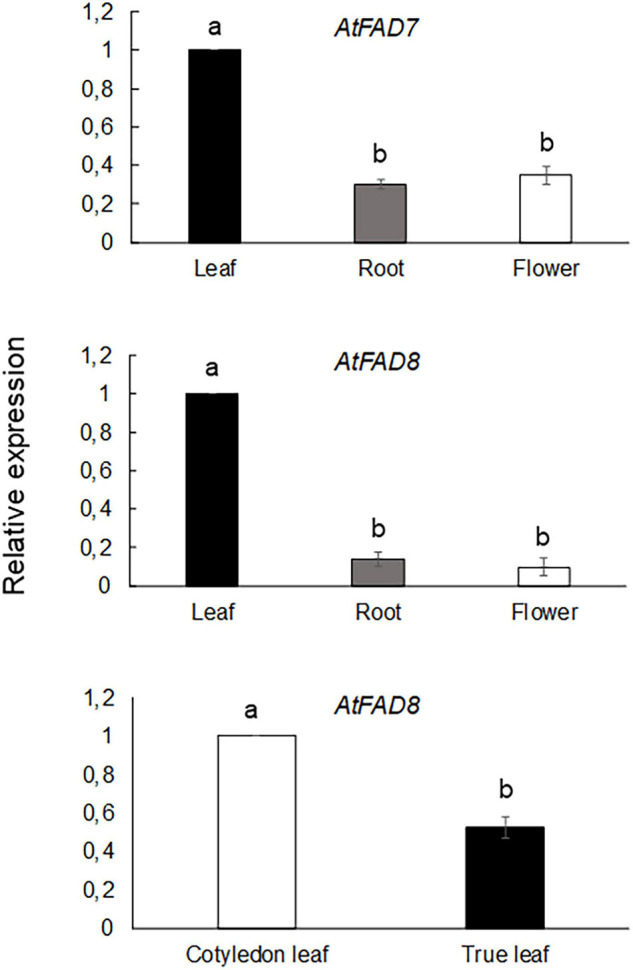
qPCR analysis of *AtFAD7* and *AtFAD8* gene expression in leaves, roots or flowers from Col-0 Arabidopsis plants grown at 22°C. *AtFAD8* gene expression from cotyledonal and true leaves from 2-week old rosette leaves is also shown in the lower panel. Data represent means from at least three biological replicates. Different letters indicate significant differences among treatments (*P* < 0.05).

qPCR analysis was performed in Col-0 plants to further confirm the results obtained with the GUS analysis. As shown in Figure 2, *AtFAD7* expression was higher in leaves when compared to that of roots or flowers, consistent with the GUS histochemical data.

To further corroborate to what extent the results obtained with histochemical localization of the deleted fragments corresponded to the elimination of specific-putative target sequences identified along the *AtFAD7* promoter sequence, site-directed mutagenesis of some specific target sequences was performed and new transgenic lines were generated to monitor GUS histochemical activity as described above. Given the results obtained in the deletion analysis, we focused our mutagenesis strategy on the MYB and WRKY putative sequences identified in the *AtFAD7* gene promoter. Mutagenesis of the three WRKY sequences identified in the *AtFAD7* promoter resulted in a drastic reduction of GUS staining in both cotyledonal and true leaves, affecting both mesophyll and leaf vasculature, when compared to control non-mutated plant lines (Figure 1C). This decrease was consistent with the results obtained with the 499 bp fragment that was devoid of two out of the three WRKY *cis*-elements detected in the *AtFAD7* promoter (Figure 1A). These results suggested that WRKY proteins might be involved in the basal expression of *AtFAD7* in leaves. Mutagenesis of the three WRKY target sequences showed a slight decrease of GUS staining in the root vasculature (Figure 1C). Interestingly, GUS staining in the root meristem was similar to that of the control 1,682 bp *AtFAD7* promoter fragment (Figure 1C). On the contrary, mutagenesis of the three WRKY sequences resulted in a complete loss of GUS staining in flowers (Figure 1C), also consistent with the results obtained in the 499 bp deletion lines (Figure 1A).

When the two putative MYB target sequences, located in the distal regions of the *AtFAD7* promoter fragment were mutated, a similar GUS staining was observed in cotyledonal and true leaves, although a slight decrease in GUS staining particularly in leaf vasculature of true leaves was observed (Figure 1C). This result was consistent with that obtained with the 703 bp deleted fragment, devoid of the two putative MYB target sites (Figure 1A). The effect of MYB mutagenesis was particularly drastic in roots and flowers, resulting in an almost complete elimination of GUS staining in the root, both in the root vasculature and at the root meristem (Figure 1C), that was even most pronounced than that observed in the 703 bp fragment (Figure 1A). In flowers, mutagenesis of the two MYB sites, eliminated the GUS staining in sepals and in the filaments of the stamen (Figure 1C), as occurred with the 703 bp fragment (Figure 1A). However, although a drastic reduction of GUS staining in the stigma of the pistil was also observed, it retained some low GUS histochemical activity (Figure 1C).

### Functional Analysis of the *AtFAD8* Gene Promoter Using Promoter:GUS Fusions

**FIGURE 3:**
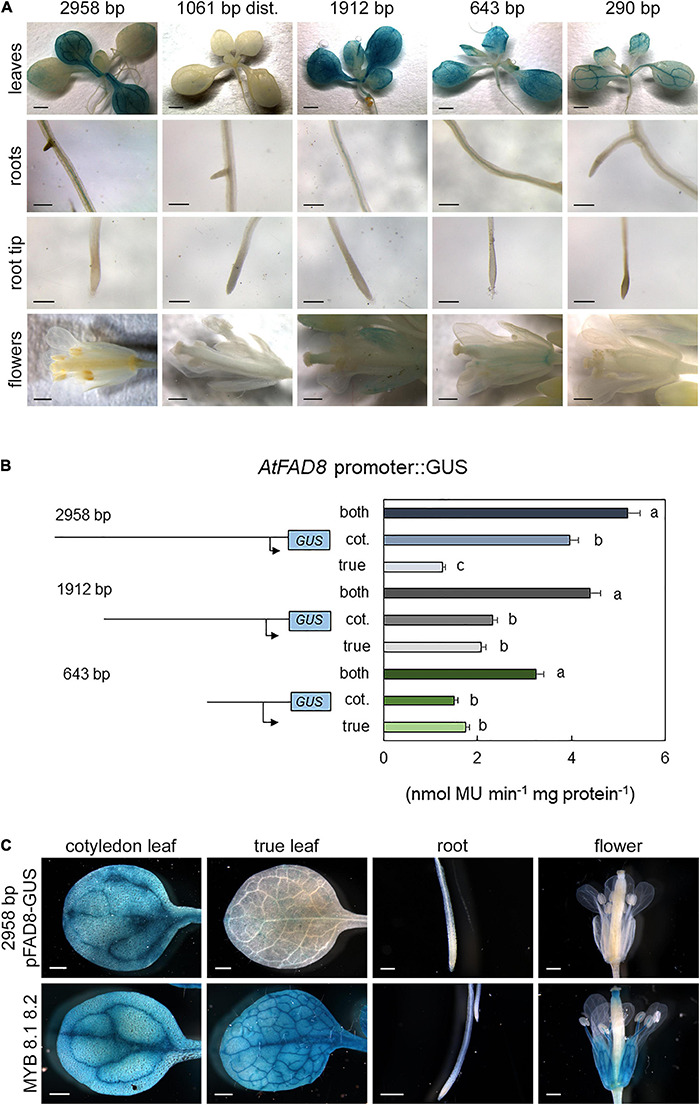
GUS histochemical localization of the *AtFAD8* gene promoter. **(A)** *AtFAD8* promoter:GUS staining in leaves, roots and flowers. Results are shown for the control *AtFAD8* 2,958 bp fragment and the four deletions of 1,061 bp, 1,912 bp, 643 bp and 290 bp, respectively. Results were obtained after 3 h of GUS staining. Images are representative of at least three independent transgenic lines. Scale bars represent: 1,000 μm in plantlets, 200 μm in roots and 500 μm in flowers. **(B)** GUS activity in the *AtFAD8* gene promoter fragments. Activity was determined in extracts from the aerial part of 2-week old plants carrying the different *AtFAD8* promoter fragments. Activity was determined separately in cotyledonal and true leaves. Results were obtained after 3 h of GUS staining. A schematic diagram of the different promoter length fragments used for the analysis is also shown. Activity was determined as nmol MU⋅min^– 1^ ⋅mg protein^– 1^. Data are means ± SE from at least five independent determinations of three independent transgenic plants. Different letters indicate significant differences among treatments (*P* < 0.05). **(C)** *AtFAD8* promoter:GUS staining in leaves, roots and flowers in transgenic lines in which the two MYB putative target sequences detected in the distal region of the *AtFAD8* gene 2,958 bp promoter fragment were modified by site-directed mutagenesis. Lines containing the 2,958 bp *AtFAD8* fragment were used as a control in all tissues. Results were obtained after3 h of GUS staining. MYB 8.1 8.2 represents the lines carrying the site-directed mutation on the MYB target sequences. Images are representative of at least three independent transgenic lines. Scale bars represent: 500 μm in leaves and flowers, 200 μm in roots.

The analysis of the *AtFAD8* gene promoter was performed on a 2,958 fragment upstream its ATG in chromosome 5 (*At5g05560*; Soria-García et al., 2019). As performed with the *AtFAD7* promoter, PLACE, PLANTCARE and MOTIFSAMPLER software analysis was performed to identify putative *cis*-regulatory elements in the *AtFAD8* promoter sequence. A putative TATA box was located near the TSP, at -195 from the ATG. As occurred in *AtFAD7*, two MYB consensus sequences (CAACTG) were located at -2,840/-2,834 and -2,708/-2,703, respectively, from the ATG (Table 1). The *AtFAD8* gene also contained two putative Myc consensus sequences (CACATG) located at -1,503/-1,498 and -652/-647, with respect to the ATG (Table 1). This target sequence was first involved in the activation of the Arabidopsis *rd22* gene in response to abiotic stress (Abe et al., 1997), and is identical to the binding-site of *ICE1*, a TF of the Myc family involved in the activation of genes in response to cold (Yamaguchi-Shinozaki and Shinozaki, 2006). Six CBF/DREB binding sites located at -1,300/-1,295, 1,297/-1,292, -346/-341, -128/-123, -125/-120, and -122/-117 with respect to the ATG were detected in the *AtFAD8* gene promoter (Table 1). It is worth mentioning that only one CBF/DREB target sequence (AGCGAC) was detected in the *AtFAD7* promoter (Table 1). The CBF/DREB target sequence contains the A/GCCGAC motif present in many drought- and cold-regulated genes (Stockinger et al., 1997; Thomashow, 1999). The presence of numerous putative CBF target sequences in the *AtFAD8* gene promoter fits well with its higher expression at low temperatures (McConn et al., 1994; Román et al., 2015). Three WRKY consensus motifs were also detected at -490/-485 (TTGACC) and -428/-423 and -309/-304 (TTGACT), also forming a cluster in the *AtFAD8* promoter sequence (Table 1). Interestingly, no G-boxes related with light regulation, were detected in the *AtFAD8* promoter sequence. As performed with *AtFAD7*, several deletions of the *AtFAD8* promoter sequence were carried out to analyze the functional relevance of the different regulatory elements identified *in silico*. *AtFAD8* promoter:GUS activity was analyzed in the different plant tissues. The 2,958 bp fragment showed a lower GUS activity (4-fold reduction) in leaves when compared with the 1,682 bp fragment used for *AtFAD7* (Soria-García et al., 2019). Therefore, it was necessary to increase GUS-staining time up to 3 h to detect *AtFAD8* promoter histochemical GUS activity. After doing so, high GUS staining was detected in cotyledonal leaves (Figure 3A). Conversely, GUS staining in true leaves was much lower (Figure 3A). GUS histochemical analysis was also performed in roots and flowers. Very low, if any, GUS staining was detected in roots from the transgenic lines harboring the 2,958 bp promoter fragment (Figure 3A). In flowers, no significant GUS staining could be observed in the transgenic lines harboring the 2,958 bp *AtFAD8* promoter fragment (Figure 3A). The results confirmed the overall lower basal promoter activity and expression of the *AtFAD8* gene when compared with its plastidial counterpart *AtFAD7* and their different tissue and organ distribution (Soria-García et al., 2019).

qPCR analysis of *AtFAD8* gene expression was also performed in Col-0 plants to compare *AtFAD8* gene endogenous expression on the different plant tissues. The qPCR analysis confirmed the GUS results showing higher *AtFAD8* expression in leaves when compared with roots or flowers where its expression was very low, consistent with the GUS results (Figure 2). qPCR analysis of *AtFAD8* gene expression in control 2-week old Col-0 plants also confirmed the different GUS histochemical pattern of *AtFAD8* in the different types of leaves, being *AtFAD8* mRNA levels in true leaves a half from those detected in cotyledonal leaves (Figure 2). This result confirmed that these differences were not an artifact of the transgene analysis.

Then, GUS staining in the different *AtFAD8* promoter deletion fragments was also monitored. No GUS staining was detected in cotyledonal or rosette leaves when the distal 1,061 bp fragment was analyzed (Figure 3A), indicating that the two putative MYB regulatory elements alone could not drive *AtFAD8* expression in leaves. However, as occurred in the case of *AtFAD7*, it cannot be discarded that some essential element for transcription could have been eliminated in this fragment. When the 1,912 bp fragment, essentially devoid of the two putative MYB target sequences, was analyzed no changes in GUS staining were observed in cotyledonal leaves. However, an increase in GUS staining in true leaves was detected, compared to that of the 2,958 bp fragment (Figure 3A). A decrease of GUS staining with respect to the 1,912 bp fragment was observed in cotyledonal and true leaves from plants harboring the 643 bp promoter deletion fragment that retained the putative WRKY and CBF/DREB elements but was essentially devoid of the two Myc motifs (Figure 3A). This decrease was even stronger in the 290 bp shortest *AtFAD8* promoter deletion fragment that retained only the putative CBF/DREB elements but was devoid of the WRKY ones (Figure 3A). This decrease was particularly relevant in mesophyll cells of cotyledonal leaves while it was less evident in leaf vasculature (Figure 3A). Finally, GUS staining was monitored in lines harboring the deleted fragments of the *AtFAD8* gene promoter in roots or flowers. No GUS staining was detected in roots, while a subtle but consistent presence of GUS staining was detected in sepals and the stigma of the pistil in the 1,912 bp deletion fragment (Figure 3A).

GUS activity in leaves determined as umbelliferol fluorescence was also monitored for the *AtFAD8* promoter. Since differences in *AtFAD8* expression and GUS staining were detected among cotyledonal and true leaves, GUS activity was monitored in the complete rosette as well as in separated cotyledonal and true leaves from 2-week old plants. Overall GUS activity for the 2,958 bp *AtFAD8* promoter sequence (5.2 nmol MU⋅min^–1^⋅mg protein^–1^) in rosette leaves was much lower (5-fold) than that obtained with the 1,682 bp *AtFAD7* promoter fragment (Figure 3B). When the activity was monitored separately, most part of the GUS activity (3.8 nmol MU⋅min^–1^⋅mg protein^–1^) corresponded to cotyledon leaves while true ones showed a much lower GUS activity (1.6 nmol MU⋅min^–1^⋅mg protein^–1^) (Figure 3B). This result was consistent with the qPCR data and the histochemical GUS staining (Figures 2, 3A). The 1,912 bp proximal fragment, essentially devoid of the two MYB elements, showed 4.06 nmol MU⋅min^–1^⋅mg protein^–1^, retaining a 78,2% of the activity of the 2,958 bp promoter sequence. Interestingly, the differences in GUS activity among cotyledonal and true leaves were substantially reduced with very similar contribution to the overall GUS activity (Figure 3A). Elimination of both putative Myc sites in the 643 bp fragment further reduced GUS activity to 2.92 nmol MU⋅min^–1^⋅mg protein^–1^, a 56,3% of that of the 2,958 bp fragment (Figure 3B). As occurred with the 1,912 bp *AtFAD8* promoter deletion, both cotyledonal and true leaves showed similar GUS activity (Figure. 3B). Further elimination of the putative WRKY cluster in the 290 bp fragment decreased GUS activity to very low values (1.77 nmol MU⋅min^–1^⋅mg protein^–1^).

As performed with the *AtFAD7* promoter, site-directed mutagenesis was carried out in some putative *cis*-acting elements identified in our previous analysis. In the case of *AtFAD8* this analysis was focused in the two putative MYB target sequences detected in the distal region of the *AtFAD8* promoter, because of their effect on GUS staining in true leaves. Mutagenesis of both MYB sites in the 2,958 bp promoter fragment resulted in a clear increase of GUS staining activity in true leaves from the mutagenized transgenic lines when compared with the control ones (Figure 3C), consistent with the results obtained with the 1,912 bp fragment (Figure 3A). Interestingly, mutagenesis of the two MYB target sites resulted not only in an increase in the GUS staining in true leaves but also in flowers (including sepals, the stigma of the pistil, and the stamen) (Figure 3C). It is worth mentioning that such an increase was detected in the 1,912 bp deletion fragment but to a much lesser extent, probably because of the absence of additional elements required for its expression that are present in the mutated one. Moreover, this GUS staining pattern was very similar to that of the 1,682 bp *AtFAD7* promoter fragment (Figure 1). These results strongly suggested that MYB TFs could be acting as repressors of *AtFAD8* gene expression under basal conditions at least in true leaves, and flowers.

### Functional Analysis of Promoter *cis*-Elements Involved in the Wound Response of *AtFAD7* Gene

**FIGURE 4:**
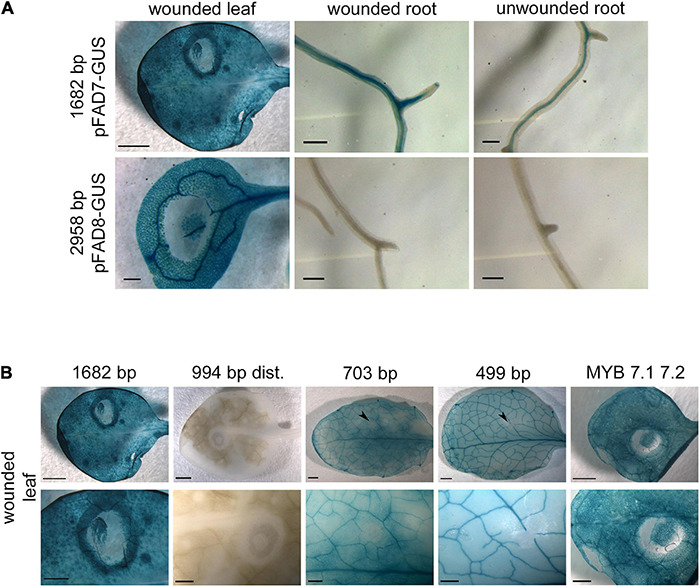
Effect of wounding on the activity of *AtFAD7* and *AtFAD8* gene promoters. **(A)** Histochemical GUS staining in wounded leaf, wounded root and unwounded root in transgenic lines carrying the 1,682 bp *AtFAD7* gene promoter fragment (upper panels) and the 2,958 bp *AtFAD8* gene promoter fragment (lower panels). Histochemical GUS assay was performed in 2-week old rosette leaves. GUS staining was performed for 1 or 3 h for *AtFAD7* and *AtFAD8* gene promoters, respectively. Images are representative of at least three independent transgenic lines. Scale bars represent 500 μm in leaves and 200 μm in roots. **(B)** Histochemical GUS localization of *AtFAD7* promoter activity after 1 h of wounding treatment and 1 h of GUS staining in the 1,682 bp control fragment, the 994 distal promoter deletion, the 703 bp and the 499 bp deletions and in transgenic lines carrying the 1,682 bp sequence in which the two MYB target sequences were modified by site-directed mutagenesis. The upper panel shows the complete leaf and the lower panel a detail of the leaf zone in which the wounded treatment was performed. MYB 7.1 7.2 represents the lines carrying the site-directed mutation on the MYB target sequences. Histochemical GUS assay was performed in 2-week old rosette leaves. Images are representative of at least three independent transgenic lines. Scale bars represent 1,000 μm for the upper panel (entire leaf) and 500 μm for the lower panel (detail).

In order to identify *cis*-regulatory elements involved in the wound response of the *AtFAD7* gene (Hamada et al., 1996; Reymond et al., 2000; Matsuda et al., 2009; Andreu et al., 2010; Soria-García et al., 2019), wound treatment was performed on different tissues in the transgenic lines harboring the 1,682 bp *AtFAD7* promoter: GUS construct and was compared to those carrying the 2,958 bp *AtFAD8* promoter:GUS one. Wounding in leaves, produced by pressing the leaf surface with a pipette tip, resulted in a strong halo of GUS staining surrounding the area pressed by the tip in the transgenic lines harboring the *AtFAD7* promoter:GUS gene (Figure 4A). An increase in GUS staining was observed in roots cut with a scalpel of these transgenic lines affecting not only the root vasculature but also the rest of the root tissue when compared to unwounded roots (Figure 4A). No effect in GUS staining was detected in wounded leaves or roots from plants carrying the 2,958 bp *AtFAD8* promoter:GUS fusion (Figure 4A), suggesting that *AtFAD8* promoter was not sensitive to wounding. This result obtained by GUS histochemical analysis was consistent to that obtained previously by qPCR (Soria-García et al., 2019).

Wounding effect was also analyzed in the transgenic lines harboring the different *AtFAD7* promoter deletions fused to GUS. Analysis of the 703 bp promoter fragment, essentially devoid of the two MYB target sequences located in the distal region of the promoter, resulted in a complete loss of the halo of GUS staining induced by wounding in leaves when compared with that obtained in the control 1,682 bp fragment (Figure 4B). Further deletion of the *AtFAD7* gene promoter (499 bp fragment) did not show any change in this pattern (Figure 4B). These results suggested that the elements that participate in the wound specific response of *AtFAD7* were located within the distal *AtFAD7* promoter region where the two MYB putative target sites were detected, upstream the 703 bp deletion, contrasting with the results obtained by Nishiuchi et al. (1999) with their 825 bp fragment. Nevertheless, the distal region of the promoter involved in the wound-specific response seemed not to be essential for its expression in leaves. In fact, in all cases, GUS staining was detected in leaf vasculature and mesophyll cells in wounded plants (Figure 1), indicating that the elements that control the basal leaf expression and the wound specific response of *AtFAD7* were different and located in different regions of the promoter.

To further confirm these results, site-directed mutagenesis was performed in both MYB putative target sequences. Mutagenesis of both MYB7.1 and MYB7.2 target sequences resulted in a substantial reduction (almost complete elimination) of the wound induced halo detected with the control 1,682 bp promoter fragment (Figure 4B). These results suggested that MYB TFs might be involved in the wound specific response of the *AtFAD7* gene.

### Functional Analysis of Regulatory Elements Involved in the Specific Hormone Responses of the *AtFAD7* and *AtFAD8* Genes

**FIGURE 5:**
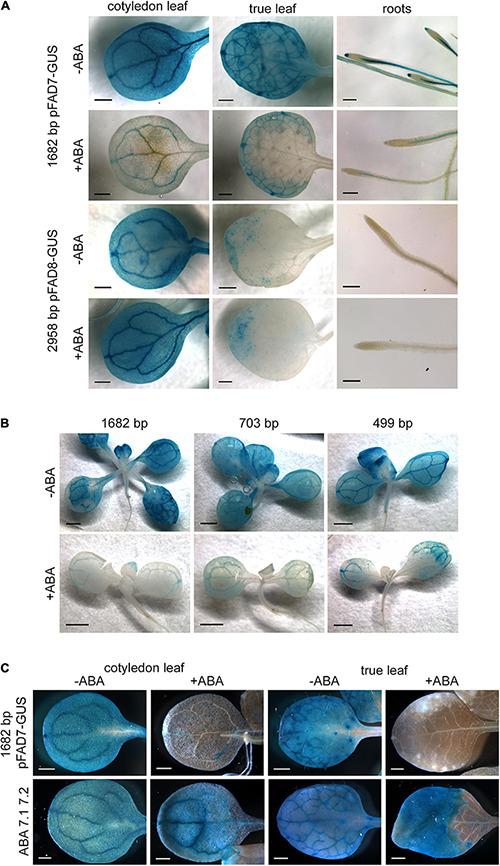
Effect of ABA on the activity of *AtFAD7* and *AtFAD8* promoters. **(A)** Histochemical GUS assay in 2-week old cotyledons, true leaves and roots from transgenic lines carrying the 1,682 bp *AtFAD7* and 2,958 bp *AtFAD8* gene promoter fragments. Two week-old plants were treated for 48 h with 100 μM ABA (+ ABA) and then subjected to 1 or 3 h of GUS incubation in the case of the 1,682 bp *AtFAD7* or the 2,958 bp *AtFAD8* gene promoter, respectively. Untreated (-ABA) plants were used as a control. Images are representative of at least three independent transgenic lines. Scale bars represent: 500 μm in leaves and 200 μm in roots. **(B)** Histochemical GUS assay in *AtFAD7* deleted promoter:GUS stable Arabidopsis lines after 48 h of 100 μM ABA treatment. Two week-old plants carrying the 1,682 bp control fragment and the 703 and 499 bp deleted fragments were used for the analysis. (+ ABA), plants treated with 100 μM ABA; (-ABA), control untreated plants. Images are representative of at least three independent transgenic lines. Scale bars represent 1,000 μm. **(C)** Histochemical GUS assay in transgenic lines in which the two ABA repression sequences detected in the *AtFAD7* 1,682 bp gene promoter were modified by site-directed mutagenesis (ABA 7.1 7.2). Two week-old plants were treated for 48 h with 100 μM ABA (+ ABA) and then subjected to 1 h of GUS incubation. The unmodified 1,682 bp *AtFAD7* gene promoter fragment was used as a control. (+ ABA), plants treated with 100 μM ABA; (-ABA), control untreated plants. Images are representative of at least three independent transgenic lines. Scale bars represent 500 μm.

We have previously reported a specific effect of MeJA and ABA on *AtFAD7* and *AtFAD8* genes, respectively at the expression level (Soria-García et al., 2019). We used the different promoter:GUS fusions to further analyze regulatory elements involved in these specific responses. MeJA treatment on lines carrying the *AtFAD8* promoter:GUS fusions did not result in significant differences with respect to untreated plants (Supplementary Figure 2). In the case of ABA, we have previously reported a strong decrease of *AtFAD7* mRNA levels in response to ABA without affecting the expression of the *AtFAD8* gene (Soria-García et al., 2019). We investigated whether this specific response was consistent with the presence of two ABA repression elements located before the TSP in the *AtFAD7* gene promoter sequence. To test this hypothesis, transgenic lines harboring the 1,682 bp *AtFAD7* and 2,958 bp *AtFAD8* promoter fragments were treated with 100 μM ABA for 48 h and then GUS histochemical analysis was performed. *ABI1* gene was used to assess the effect of ABA in our experiments (Soria-García et al., 2019). In the transgenic lines carrying the 1,682 bp promoter fragment fused to GUS, ABA treatment resulted in a drastic reduction of the GUS staining in cotyledonal and true leaves, and in the root (Figure 5A). No changes in the GUS histochemical pattern upon ABA treatment were observed in the transgenic lines harboring the 2,958 bp *AtFAD8* promoter fragment either in leaves or roots (Figure 5A). These results were consistent with our previous qPCR data (Soria-García et al., 2019), confirming that the effect of ABA was *AtFAD7* specific.

The effect of ABA was also analyzed in the transgenic lines harboring the different deleted fragments of the *AtFAD7* gene promoter. Strong decrease of GUS staining was detected in the 703 and 499 bp promoter deleted fragments upon ABA treatment (Figure 5B). It is worth mentioning that the two putative ABA repression elements detected at – 278/-267 and -203/-197 with respect to the ATG were still present in these deleted fragments and, therefore, the putative ABA repression sequences could still be operative. To further confirm their functionality, site-directed mutagenesis was performed in both ABA elements of the 1,682 bp *AtFAD7* promoter fragment and new stable transgenic lines were obtained and further analyzed. After 48 h treatment with 100 μM ABA, transgenic lines expressing the promoter in which both ABA sequences have been mutated showed GUS staining in cotyledonal and true leaves (Figure 5C), although in true leaves, a portion of the leaf close to the peduncle showed no staining.

### Identification of Similar *cis*-Acting Elements in *FAD7* and *FAD8* Genes From Other Plant Species

**FIGURE 6:**
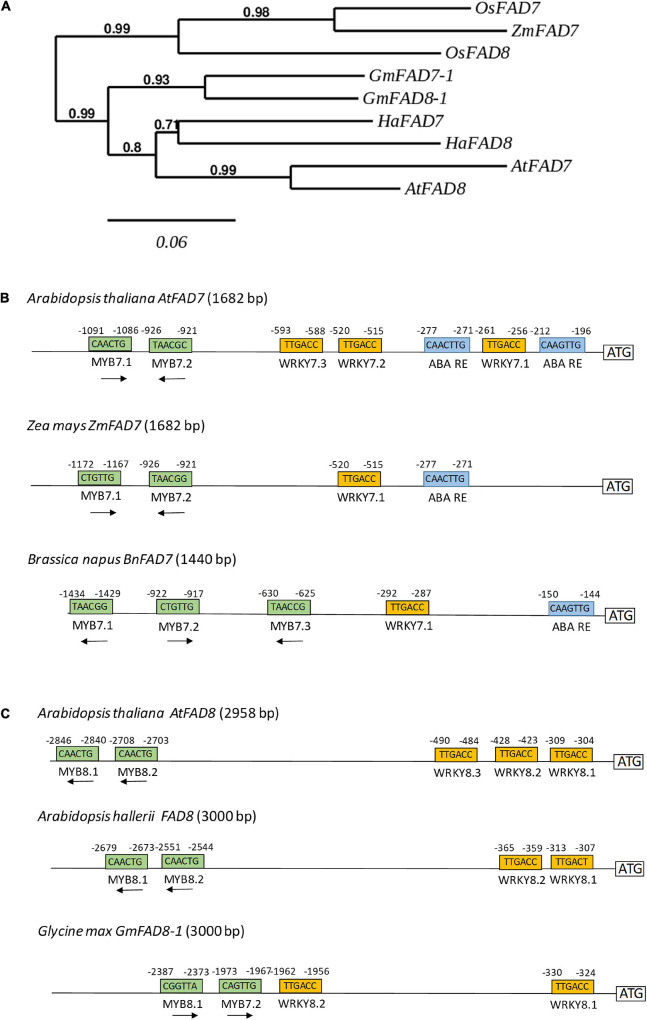
*In silico* analysis of the presence of putative transcription factors identified in the *AtFAD7* and *AtFAD8* in promoter sequences from other plant species. **(A)** Phylogenetic tree of plastidial ω-3 fatty acid desaturases FAD7 and FAD8 from Arabidopsis, soybean, sunflower, maize and rice. Protein sequences obtained at the phytozome database were subjected to a CLUSTALW multiple alignment and phylogeny was analyzed by the PHYML software with bootstrap 500. **(B)** *In silico* comparative analysis of WRKY, MYB and ABA repression motifs in the *FAD7* gene promoter from *Zea mays* (*ZmFAD7*, 1,682 bp) and *Brassica napus* (*BnFAD7*, 1,440 bp). **(C)** *In silico* comparative analysis of WRKY and MYB motifs in the *FAD8* gene promoter from *Arabidopsis hallerii* (*AhFAD8*, 3,000 bp) and *Glycine max* (*GmFAD8-1*, 3,000 bp). All genomic sequences were retrieved from Phytozome database. Relative position with respect to the ATG is indicated in small number in each box.

At this point we analyzed whether some of the *cis*-regulatory elements identified in the functional analysis of both *AtFAD7* and *AtFAD8* promoters could be also detected in the same promoters from other plant species. Phytozome database was used to retrieve the sequences upstream both genes in different plant species. PLACE, PLANTCARE and MOTIFSAMPLER analysis software were used for the detection of *cis*-acting elements on these sequences. A phylogenetic tree was performed to analyze to what extent the sequences analyzed were orthologs or not. The tree showed a clear separation of the *FAD7* and *FAD8* genes between monocots (rice, maize) and dicots (Arabidopsis, soybean, sunflower), Figure 6A. The results showed an aggrupation of FAD7 and FAD8 proteins into each plant species rather than into the desaturase type (Figure 6A). A similar conclusion was obtained when we analyzed the soybean ω-3 plastidial fatty acid desaturase multigene family (Andreu et al., 2010). This observation might suggest that plastidial ω-3 fatty acid desaturase genes might have originated by gene duplication in each species. Results obtained through the analysis of the desaturase multigene family in *Brassica napus* might support this hypothesis (Scheffer et al., 1997). This might explain the presence of putative MYB regulatory sequences in the distal regions or WRKY clusters in the proximal ones in both promoters in Arabidopsis. Analyzing FAD genes separately, two MYB target sequences, placed at -1,167 and -921 from the ATG were detected in the *Zea mays ZmFAD7* (1,682 bp) promoter (Figure 6B). In *Brassica napus BnFAD7* (1,440 bp) three MYB target sequences were detected located at -1,429, -917, and -625, with respect to the ATG (Figure 6B). It is worth mentioning that in both cases, MYB7.1 and MYB7.2 were located in almost identical position with respect to that of *AtFAD7* (Figure 6B). Search for WRKY target sequences resulted in a single WRKY box (WRKY 7.1), not a cluster, in both the *ZmFAD7* (-515) and the *BnFAD7* (-287) promoters. These sequences were located again in almost identical positions with respect to those found in the *AtFAD7* gene promoter (Figure 6B). Finally, single ABA repression sequences were detected at -271 and -144 with respect to the ATG in the *ZmFAD7* and *BnFAD7* gene promoters, respectively (Figure 6B).

As occurred with the *AtFAD8* gene promoter, two MYB target sequences were detected at -2,679 and -2,551 bp with respect to its ATG in the *Arabidopsis hallerii AhFAD8* (3,000 bp) gene promoter and at -2,387 and -1,973 bp, respectively, in the case of *Glycine max GmFAD8-1* (3,000 bp) gene promoter (Figure 6C). In both cases, the position of these MYB sites was very similar to that of the *AtFAD8* gene promoter. Two WRKY target sequences were detected in the *AhFAD8* gene at -365 and -313 bp, in almost identical positions with respect to those in the *AtFAD8* gene (Figure 6C). In the case of the *GmFAD8-1* gene, another two WRKY sequences were found at -1,962 and -330 bp, but in this case, only one of them was located in a similar position with respect to that in the *AtFAD8* gene promoter (Figure 6C).

## Discussion

Over the approximately 1,800 total TFs detected in the Arabidopsis genome, only two, WRI1 and bZIP67, have been linked to the regulation of acyl-lipid metabolism in the seed (Cernac and Benning, 2004; Baud et al., 2007; Mendes et al., 2013). Similarly, MYB89 and MYB96 have been involved in the regulation of genes that encode proteins participating in fatty acid biosynthesis during seed development (Wang et al., 2014; Li et al., 2017; Lee et al., 2018). This knowledge of the control of fatty acid biosynthesis in seeds contrasts with the limited information about fatty acid biosynthesis, and particularly TAs, in other tissues like leaves, where they represent an 80% of total fatty acids constituents of bulk membrane lipids. Furthermore, leaf TAs are directly involved in cold-acclimation (Iba, 2002) or act as precursors of JA in defense responses (Farmer et al., 2003). In flowers, JA synthetized from TAs is essential for pollen maturation (McConn and Browse, 1996). The position of TAs at the basis of many developmental and stress signaling pathways, implies a tight regulation of desaturase expression through the action of unknown TFs. Unfortunately, the identification of TFs through the screening of mutant phenotypes is usually difficulted by the redundant function of regulators (McGlew et al., 2015) or target genes, as occurs with the desaturases, particularly FAD7 and FAD8, with similar activity and compensatory responses (Román et al., 2015). To overcome this problem, we developed a strategy based in the functional dissection of both *AtFAD7* and *AtFAD8* promoter sequences and GUS reporter analysis directed toward the identification of *cis*-regulatory elements essential for the expression of *AtFAD7* and *AtFAD8* genes in different plant tissues and organs and in response to specific hormone or stress treatments.

Our data showed high *AtFAD7* promoter:GUS activity not only in leaves, where it was very high as expected, but also in non-photosynthetic tissues like flowers or roots. Moreover, our data revealed a tissue-expression pattern in roots and flowers, with specific histochemical activity in the stamen and the stigma of the pistil in flowers or in the root vasculature and meristem (Figure 1). Expression of *AtFAD7* in leaves and flowers is consistent with the role of TAs in membrane lipids or in pollen maturation, respectively (Román et al., 2015; McConn and Browse, 1996). Its expression in the root vasculature and meristem is intriguing. Although TAs levels in roots are not very high, they could act as precursors of JA, which is a negative effector of root length (Staswick et al., 1992). In addition, growing roots penetrate in the soil and, accordingly, sense a mechanical stress that could induce JA biosynthesis, requiring TA precursors. The results obtained through the functional dissection of the *AtFAD7* promoter or the mutagenesis of specific target sequences, suggested that different elements and regulatory pathways are behind this tissue and organ specific expression pattern. Thus, in leaves, members of the WRKY TF family seemed to be essential for the basal expression of *AtFAD7* since elimination of the W boxes either in the deleted promoter fragments or through site-directed mutagenesis drastically reduced *AtFAD7* promoter:GUS histochemical activity in leaves (Figure 1). In non-photosynthetic tissues, like flowers or roots the role of these *cis*-acting elements seemed to be different. In flowers, both WRKY and MYB *cis*-acting elements seemed to be necessary for the expression of the *AtFAD7* gene since elimination by site-directed mutagenesis of the two MYB sequences or the WRKY cluster, produced independently an almost complete loss of GUS histochemical activity in the stamen of the anthers and the pistil of the ovarium (Figure 1). On the contrary, in roots, the role of the two MYB sites alone seemed to be essential since the mutagenesis of the two MYB sites resulted in a complete loss of GUS activity (Figure 1) while elimination of three WRKY sequences did not diminish GUS histochemical activity in the root meristem or vasculature. Although the major role of WRKYs has been related to plant responses to pathogens, their expression in numerous cell types and under different physiological conditions indicated that WRKYs participate in a wide variety of biological processes including senescence (Miao et al., 2004), plant growth (Yu et al., 2012), and plant development (Rushton et al., 2010; Yu et al., 2012), that might help to understand the role of WRKYs in the basal expression of *AtFAD7*, particularly in leaves. On the contrary, the involvement of MYB TFs in the control of the expression of *AtFAD7* in the root, particularly in the root meristem, is less surprising. Thus, MYB36 regulates genes required for Casparian strip formation and differentiation both in the primary and lateral roots (Kamiya et al., 2015; Liberman et al., 2015; Fernández-Marcos et al., 2016). Furthermore, MYB59 has been involved in root development by altering mitosis in the root tip cells (Mu et al., 2009). Mutagenesis of the two MYB target sequences in flowers resulted in a complete loss of the *AtFAD7* promoter-GUS staining pattern in the stigma of the ovarium and the filament of the stamen. Two MYB TFs, MYB21 and MYB24, have been shown to participate in JA-regulated stamen development in Arabidopsis (Song et al., 2011). Interestingly, complementation of MYB21 alone in a *coi1*-1 background partially restored male fertility but not JA-regulated root growth. The double mutant *myb21myb24* showed a more drastic phenotype suggesting that both MYB21 and MYB24 proteins function redundantly for the control of anther dehiscence and pollen maturation in Arabidopsis (Song et al., 2011). This could be consistent with the presence of two MYB target sequences in the *AtFAD7* gene.

Interestingly, the role of these two putative MYB target sequences present in the *AtFAD7* gene promoter was not restricted to the basal expression of *AtFAD7* in non-photosynthetic tissues. Elimination of these two MYB sites in the promoter-deleted fragments or in the site-directed MYB mutants, induced a substantial loss of the wound responsive pattern observed with the 1,682 bp *AtFAD7* gene promoter fragment in leaves (Figure 4) without affecting its basal expression, suggesting that MYB TFs were specifically involved in the wound-response of *AtFAD7*. Transcriptomic analysis of wound-responsive genes in Arabidopsis has revealed that several members of the MYB family like *At*MYB15, *At*MYB34, *At*MYB51 and *At*MYB75 were associated to the wound response or resistance to herbivore attack (Cheong et al., 2002; Devoto and Turner, 2003; Taki et al., 2005). However, since MYB TFs are a multigene family, with many redundant and compensatory phenotypes (Zhu, 2016), it is very difficult to attribute to a specific MYB protein the specific control of *AtFAD7* in these responses. It is worth mentioning that the two MYB target sequences involved in the wound response of *AtFAD7* were located in the distal region of the promoter, at -1,093/-1,087 and -927/-922 with respect to the ATG, upstream the 703 bp deletion (Table 1). These results contrasted with previous data that identified two regions in the *AtFAD7* promoter responsible of the different wound-response in leaves (-259/-198) or roots (-521/-360), Nishiuchi et al. (1999). Both regions fell within the WRKY cluster identified in our analysis. Our results do not support an involvement of sequences in that region in the wound response (Figure 4). However, since they participate in the basal expression of *AtFAD7*, a possible interaction between MYB and WRKYs upon wounding cannot be precluded. Such interaction has been already reported between MYB44 and WRKY70 for the coordination of salicylic acid (SA) and JA-dependent defense responses in Arabidopsis (Shim et al., 2013).

We previously reported that both *At*FAD7 and *At*FAD8 plastidial ω-3 desaturases showed differences in protein relative abundance and transcript levels in leaves (Soria-García et al., 2019). The higher specificity of FAD8 for 18:2 substrates associated to PG or sulfolipids, which represent minor lipid classes in plastid membranes (Román et al., 2015) may account for these differences. In this work, we have detected a differential basal expression pattern of *AtFAD8* between cotyledonal and true leaves (Figures 2, 3). The differences in *AtFAD8* expression between both types of leaves are not striking since they possess partly independent developmental programs (Chandler, 2008). These results suggest that at least in leaves, the coordination between *At*FAD7 and *At*FAD8 plastidial ω-3 desaturases is tightly regulated to maintain an appropriate unsaturation level of membrane lipids. Our data indicate that MYB TFs could be behind the control of that regulation. Elimination of the two MYB sequences present in the distal region of the *AtFAD8* 2,958 bp promoter fragment resulted in an increase of the GUS histochemical activity in leaves and flowers without great changes in cotyledonal leaves (Figure 3). Interestingly, site-directed mutagenesis of the two distant MYB target sequences resulted in a GUS histochemical pattern almost identical to that obtained with the *AtFAD7* 1,682 bp promoter fragment in leaves or flowers (Figures 1A, 3A). These results altogether strongly suggest that MYB TFs are acting as repressors of *AtFAD8* gene expression in the whole plant and modulating the specific contribution of *AtFAD8* to the total plastidial ω-3 desaturase activity. Under normal growth conditions or under circumstances in which high *AtFAD7* activity is required (i.e., like wounding or defense responses), *AtFAD8* expression was low and much less abundant than *AtFAD7* (Román et al., 2015; Soria-García et al., 2019). Only when an increase of a specific *At*FAD8 plastidial ω-3 desaturase activity is required, as it occurs under cold exposure, this repressor effect would be released (Román et al., 2015; Soria-García et al., 2019). This might help to explain the opposite regulations to biotic and abiotic stresses of both ω-3 plastidial desaturase genes. Thus, *AtFAD7* is induced by wounding or pathogen attack, while *AtFAD8* is insensitive to these defense responses (Román et al., 2015; Soria-García et al., 2019). Similarly, *At*FAD8 was cold up-regulated while *At*FAD7 seemed to be cold down-regulated (McConn et al., 1994; Berberich et al., 1998; Román et al., 2015). In this context, the ABA-dependent regulation of *AtFAD7* is particularly interesting. ABA is one of the major hormones involved in abiotic stress, particularly in cold-response and acclimation (Yamaguchi-Shinozaki and Shinozaki, 2005). We already reported an ABA-specific repression of *AtFAD7* gene that could be consistent with the presence of the two ABA repression elements detected in its promoter (Soria-García et al., 2019). The functional analysis of the *AtFAD7* promoter deletions as well as the site-directed mutagenesis lines suggested that both sequences were functional (Figure 5). These ABA repression sequences could be involved in the negative regulation of *At*FAD7 plastidial ω-3 desaturase activity to favor that of *At*FAD8 under conditions, such as cold, where *At*FAD8 activity is preferred to maintain the activity of the photosynthetic complexes in the plastid membrane (Román et al., 2015; Soria-García et al., 2019).

## Conclusion

Our strategy based in the functional dissection of the promoters from the *AtFAD7* and *AtFAD8* genes, has provided a first picture of the *cis*-regulatory elements and promoter regions involved in the coordination of basal plastidial *AtFAD7* and *AtFAD8* ω-3 desaturase expression in tissues other than seed, where TAs are more abundant, revealing different regulatory pathways behind the specific tissue and organ expression pattern of both plastidial desaturases. In addition, *cis*-regulatory elements involved in their specific wound and ABA responses have also been identified. Our data may explain the differences in relative abundance or response to biotic and abiotic stresses of both desaturases. Further effort will be directed toward the identification of the specific TFs involved in these regulations.

## Data Availability Statement

The original contributions presented in the study are included in the article/Supplementary Material, further inquiries can be directed to the corresponding author/s.

## Author Contributions

ML and ÁS-G performed the experiments corresponding to the GUS analysis of the different transgenic lines including the deleted and mutagenized promoter fragments in the different tissues and treatments. AC generated the deleted fragments of the promoters and performed the cloning in the GUS vector. PL helped in the characterization of the transgenic lines and participated in the RNA and cDNA extractions as well as qPCR analysis. MR helped in the design of the GUS constructs and provided expertise in the GUS histochemical analysis and optical microscopy. RP helped in the design of the experiments and discussion of the results. MA conceived and designed the study, participated in some GUS activity experiments, and prepared the manuscript. All authors discussed, commented and approved the final version of the manuscript.

## Conflict of Interest

The authors declare that the research was conducted in the absence of any commercial or financial relationships that could be construed as a potential conflict of interest.

## Publisher’s Note

All claims expressed in this article are solely those of the authors and do not necessarily represent those of their affiliated organizations, or those of the publisher, the editors and the reviewers. Any product that may be evaluated in this article, or claim that may be made by its manufacturer, is not guaranteed or endorsed by the publisher.

## Footnotes

1
http://bioinformatics.intec.ugent.be/MotifSuite/motifsampler.php

2
http://www.dna.affrc.go.jp/PLACE

3
http://bioinformatics.psb.ugent.be/webtools/plantcare.html

4
http://bioinformatics.intec.ugent.be

## Supplementary Material

Supplementary Figure 1 GUS histochemical activity in seeds from Arabidopsis transgenic lines expressing the 1,682 bp *AtFAD*7 promoter:GUS fusión **(A)**, the 2,958 bp *AtFAD8* promoter:GUS fusión **(B)** and the empty vector **(C)**. A detail photograph of the extremes of the pod in A is also shown. Bars represent 500 μm size.

Supplementary Figure 2 **(A)** Effect of 100 μM MeJA on the GUS histochemical staining in 2-week old plants from transgenic lines carrying the *AtFAD8* 2,958 bp promoter fragment and the 1,912 bp, 643 bp and 290 bp deleted promoter fragments, respectively. Upper panel shows control untreated plants. Lower panel shows plants treated with 100 μM MeJA for 2 h. Results were obtained after 3 h of GUS staining. Images are representative of at least three independent transgenic lines. Scale bars represent: 1,000 μm in plantlets. **(B)** Effect of 100 μM MeJA on the GUS histochemical staining in cotyledonal and true leaves from 2-week old plants carrying the control *AtFAD8* 2,958 bp promoter fragment (upper panel) and transgenic lines in which the two putative distal MYB target sequences were modified by site-directed mutagenesis (lower panel). Results were obtained after 3 h of GUS staining. MYB 8.1 8.2 represents the lines carrying the site-directed mutation on the MYB target sequences. Images are representative of at least three independent transgenic lines. Scale bars represent: 500 μm in leaves.

Supplementary Table 1 List and names of PCR primers used in this study.
