# Supplementary material for: Different Cis-Regulatory Elements Control the Tissue-Specific Contribution of Plastid ω-3 Desaturases to Wounding and Hormone Responses
Source: Front Plant Sci. 2021 Oct 27;12:727292. doi: 10.3389/fpls.2021.727292 (PMC8578140; doi:10.3389/fpls.2021.727292)
Supplement: Supplementary Figure 1 — GUS histochemical activity in seeds from Arabidopsis transgenic lines expressing the 1,682 bp AtFAD7 promoter:GUS fusión (A), the 2,958 bp AtFAD8 promoter:GUS fusión (B) and the empty vector (C). A detail photograph of the extremes of the pod in A is also shown. Bars represent 500 μm size. [file Data_Sheet_1.PDF]

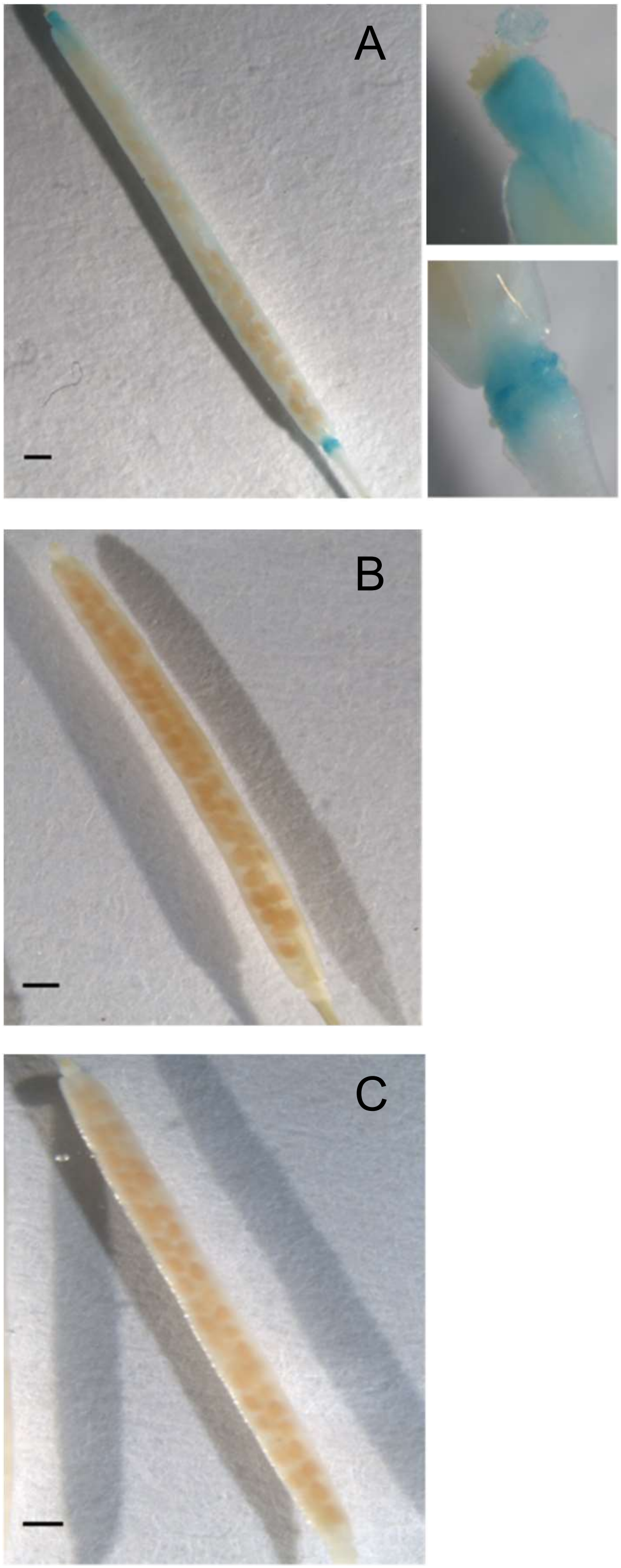

Supplementary Figure 1. GUS histochemical activity in seeds from Arabidopsis transgenic lines expressing the 1682 bp *AtFAD*7 promoter::GUS fusión (A), the 2958 bp *AtFAD8* promoter ::GUS fusión (B) and the empty vector (C). A detail photograph of the extremes of the pod in A is also shown. Bars represent 500 µm size.
