# Supplementary figures and images for: Different Cis-Regulatory Elements Control the Tissue-Specific Contribution of Plastid ω-3 Desaturases to Wounding and Hormone Responses
Source: Front Plant Sci. 2021 Oct 27;12:727292. doi: 10.3389/fpls.2021.727292 (PMC8578140; doi:10.3389/fpls.2021.727292)

A

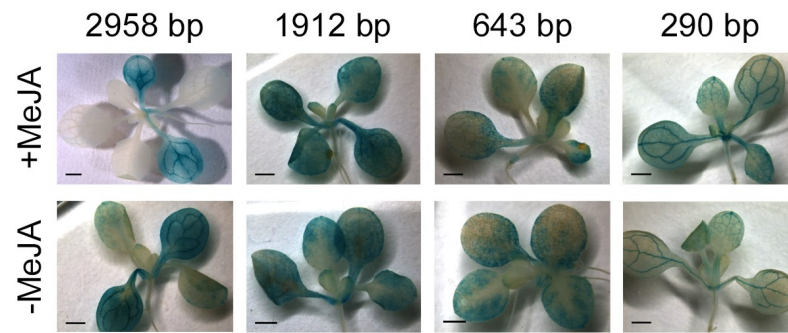

B

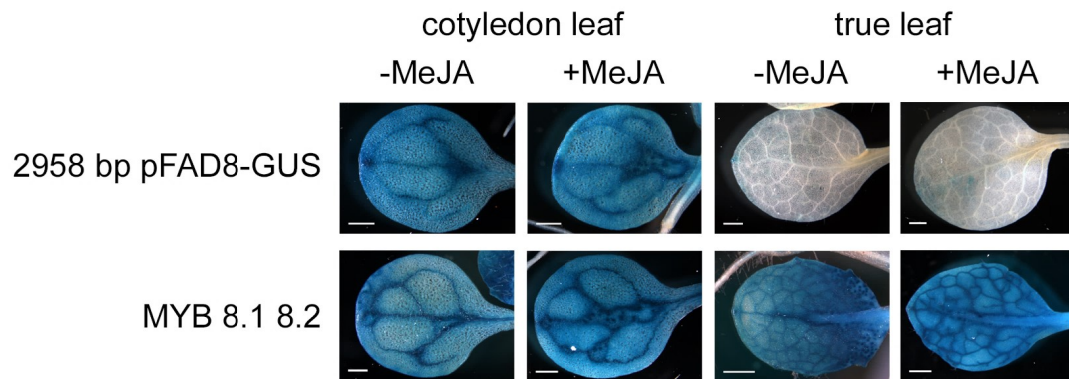

Supplement: Supplementary Figure 2 — (A) Effect of 100 μM MeJA on the GUS histochemical staining in 2-week old plants from transgenic lines carrying the AtFAD8 2,958 bp promoter fragment and the 1,912 bp, 643 bp and 290 bp deleted promoter fragments, respectively. Upper panel shows control untreated plants. Lower panel shows plants treated with 100 μM MeJA for 2 h. Results were obtained after 3 h of GUS staining. Images are representative of at least three independent transgenic lines. Scale bars represent: 1,000 μm in plantlets. (B) Effect of 100 μM MeJA on the GUS histochemical staining in cotyledonal and true leaves from 2-week old plants carrying the control AtFAD8 2,958 bp promoter fragment (upper panel) and transgenic lines in which the two putative distal MYB target sequences were modified by site-directed mutagenesis (lower panel). Results were obtained after 3 h of GUS staining. MYB 8.1 8.2 represents the lines carrying the site-directed mutation on the MYB target sequences. Images are representative of at least three independent transgenic lines. Scale bars represent: 500 μm in leaves. [file Data_Sheet_2.pdf]
