# Supplementary material for: Different Cis-Regulatory Elements Control the Tissue-Specific Contribution of Plastid ω-3 Desaturases to Wounding and Hormone Responses
Source: Front Plant Sci. 2021 Oct 27;12:727292. doi: 10.3389/fpls.2021.727292 (PMC8578140; doi:10.3389/fpls.2021.727292)
Supplement: Supplementary Table 1 — List and names of PCR primers used in this study. [file Table_1.DOCX]

**Supplementary Table 1**. List of primers used in this study.

*Primers used for amplification of the AtFAD7 promoter fragments*

FAD7p 1682bp_F CACCCTCTCAGATCTCTCTCCATCGTT

FAD7p 1682bp_R TAGAACTTGAGCTCTCTCCCCAGA

FAD7p 994bp distal_F GCCTGAAATAAATTAGCCCAAGAG

FAD7p 703bp_F CACCGGGCTAATTTATTTCAGGCTGA

FAD7p 499bp_F CACCGTTTCTTCACCAAACTCCTCTTG

*Primers used for site-directed mutagenesis of AtFAD7 putative target sites*

MYB7.1 mut_F

CGTTCTCTGATTCCGCATGAATTTCTTTGGGGGGGGGGAGAAGAAAATCACAAATAAGAGGC

MYB7.1 mut_R

GCCTCTTATTTGTGATTTTCTTCTCCCCCCCCCCAAAGAAATTCATGCGGAATCAGAGAACG

MYB7.2 mut_F

GGTCCTTAATCGCGCTGGTAAATTAAAGATTGGGGGGTGATTATCCTTTTTTTTTTTCTTTTCTACAGTCTTT

MYB7.2 mut_R

AAAGACTGTAGAAAAGAAAAAAAAAAAGGATAATCACCCCCCAATCTTTAATTTACCAGCGCGATTAAGGACC

ABA7.1 mut_F

GATTTTTTTTTGTGACGGTCAATTTAGACAGGGGGGGAGGTTTCAACACGGGTTTGATTGATCTCG

ABA7.1 mut R

CGAGATCAATCAAACCCGTGTTGAAACCTCCCCCCCTGTCTAAATTGACCGTCACAAAAAAAAATC

ABA7.2 mut_F

GTTCGCACGCTTATGTCTTATATAAGTTAGCTAGGGGGGGAAAGGAGAAGGAAATTTATTAGTGATGGTGTG

ABA7.2 mut R

CACACCATCACTAATAAATTTCCTTCTCCTTTCCCCCCCTAGCTAACTTATATAAGACATAAGCGTGCGAAC

WRKY 7.1 mut_F

CAATAGGGACAAACCAAATCTCATGCTACTGGGGGGGTTACATGGACGGTGAACATAAAAATTGAA

WRKY 7.1 mut_R

TTCAATTTTTATGTTCACCGTCCATGTAACCCCCCCAGTAGCATGAGATTTGGTTTGTCCCTATTG

WRKY 7.2 mut_F

AGTTTGGTGAAGAAACCTATTTGAGAATTTGGGGGGGTTACGTTTCAATGTTTCACACCCTATACCTA

WRKY 7.2 mut_R

TAGGTATAGGGTGTGAAACATTGAAACGTAACCCCCCCAAATTCTCAAATAGGTTTCTTCACCAAACT

WRKY 7.3 mut_F

GATGTGAGATTTTTTTTTGTGACGGGGGGTTTAGACACAAGTTGAGGTTTCAACACGGG

WRKY 7.3 mut_R

CCCGTGTTGAAACCTCAACTTGTGTCTAAACCCCCCGTCACAAAAAAAAATCTCACATC

*Primers used for amplification of the AtFAD8 promoter fragments*

FAD8p 2958kb_F CACCACATTGGTGTGCCGTCAGTA

FAD8p 2958kb_R TGGAGCCTCTTCCCAAGAA

FAD8p 1061bp distal_F CACCACATTGGTGTGCCGTCAGTA

FAD8p 1912bp_F CACCCTGGTGAGATTTCTTAGAAGAGAGC

FAD8p 643bp_F CACCCTTGCTAAGCAAATGGAGAAGC

FAD8p 290bp_F CACCCCTAAATCCCGCTTTATTCCTTC

*Primers used for site-directed mutagenesis of AtFAD8 putative target sites*

MYB8.1 mut_F

CTCCAACAGGCTCAAAAAGTGATCCCCCCCATCACAACTCCATAAATTCTGAAGA

MYB8.1 mut_R

TCTTCAGAATTTATGGAGTTGTGATGGGGGGGATCACTTTTTGAGCCTGTTGGAG

MYB8.2 mut _F

TGCCTGAGACCTCTAAAGAACCCCCCCCCGATTTGTATGCACATTATAGGT

MYB8.2 mut_R

ACCTATAATGTGCATACAAATCGGGGGGGGGTTCTTTAGAGGTCTCAGGCA

*Monitorization of hormone effect*

ABI1_F ATGGAGGAAGTATCTCCGGCGATC

ABI1_R TCAGTTCAAGGGTTTGCTCTTG

LOX2_F ATGTATTGTAGAGAGTCCTTGTCGA

LOX2_R TCAAATAGAAATACTATAAGGAACACCC

| *qPCR* |  |
| --- | --- |
| qEF1α_F | TGAGCACGCTCTTCTTGCTTTCA |
| qEF1α_R | GGTGGTGGCATCCATCTTGTTACA |
| qFAD7_F | CTCTCCAACAACAACAAATTCAGAC |
| qFAD7_R | CCAAAAGACAGAGGAGATGATGAT |
| qFAD8_F | GCCTCTAACCCTAAACCCA |
| qFAD8_R | CGGGAATTGAGAAGAGAAGAA |
